# Long‐term Outcome of Isobar TTL System for the Treatment of Lumbar Degenerative Disc Diseases

**DOI:** 10.1111/os.14025

**Published:** 2024-03-06

**Authors:** Junhu Li, Qiujiang Li, Zhipeng Deng, Linnan Wang, Lei Wang, Yueming Song

**Affiliations:** ^1^ Department of Orthopedic Surgery and Orthopedic Research Institute, West China Hospital and West China School of Medicine Sichuan University Chengdu China

**Keywords:** Adjacent segment degeneration, Dynesys, Isobar TTL, lumbar degenerative disc diseases, lumbar spondylolisthesis

## Abstract

**Objective:**

The Isobar TTL dynamic fixation system has demonstrated favorable outcomes in the short‐term treatment of lumbar degenerative disc diseases (LDDs). However, there is a paucity of extensive research on the long‐term effects of this system on LDDs. This study aimed to evaluate the long‐term clinical and radiological outcomes of patients with LDDs who underwent treatment utilizing the Isobar TTL dynamic fixation system.

**Methods:**

The study analyzed the outcomes of 40 patients with LDDs who underwent posterior lumbar decompression and received single‐segment Isobar TTL dynamic internal fixation at our hospital between June 2010 and December 2016. The evaluation of clinical therapeutic effect involved assessing postoperative pain levels using the visual analogue scale (VAS) and Oswestry disability index (ODI), both before surgery, 3 months after, and the final follow‐up. To determine the preservation of functional motion in dynamically stable segments, we measured the range of motion (ROM) and disc height of stabilized and adjacent segments preoperatively and during the final follow‐up. Additionally, we investigated the occurrence of adjacent segment degeneration (ASD).

**Results:**

Forty patients were evaluated, with an average age of 44.65 years and an average follow‐up period of 79.37 months. Fourteen patients belonged to the spondylolisthesis group, while the remaining 26 were categorized under the stenosis or herniated disc group. The preoperative ROM of the stabilized segment exhibited a significant reduction from 8.15° ± 2.77° to 5.00° ± 1.82° at the final follow‐up (*p* < 0.001). In contrast, there was a slight elevation in the ROM of the adjacent segment during the final follow‐up, increasing from 7.68° ± 2.25° before surgery to 9.36° ± 1.98° (*p* < 0.001). The intervertebral space height (IH) in the stabilized segment exhibited a significant increase from 10.56 ± 1.99 mm before surgery to 11.39 ± 1.90 mm at the one‐week postoperative follow‐up (*p* < 0.001). Conversely, there was a notable decrease in the IH of the adjacent segment from 11.09 ± 1.82 mm preoperatively to 10.86 ± 1.79 mm at the one‐week follow‐up after surgery (*p* < 0.001). The incidence of ASD was 15% (6/40) after an average follow‐up period of 79.37 months, with a rate of 15.38% (4/26) in the stenosis or herniated disc group and 14.29% (2/14) in the spondylolisthesis group; however, no statistically significant difference was observed in the occurrence of ASD among these groups (*p* > 0.05).

**Conclusion:**

The Isobar TTL dynamic fixation system is an effective treatment for LDDs, improving pain relief, quality of life (QoL) and maintaining stabilized segmental motion. It has demonstrated excellent long‐term clinical and radiographic results.

## Graphical abstract

Patients treated with posterior lumbar decompression and single‐segment Isobar TTL dynamic internal fixation were followed up for a mean of 79.37 months. The preoperative, at 3 months, and final follow‐up postoperative visual analogue scale (VAS) back and leg pain scores and Oswestry disability index (ODI) scores were observed and recorded to assess the clinical therapeutic effect. The range of motion (ROM) and the disc heights of stabilized and adjacent segments were measured preoperatively and at the last follow‐up to evaluate the preservation of functional movement in the dynamically stabilized segment. The results show that the treatment of LDDs with the Isobar TTL dynamic fixation system is effective in pain relief, QoL, and maintenance of stabilized segment lumbar ROM, showing good long‐term clinical and radiographic results.

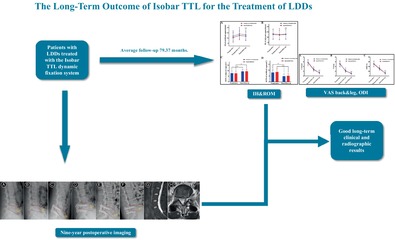

## Introduction

Lumbar interbody fusion is widely acknowledged as the preferred approach for managing various lumbar degenerative diseases, such as disc degeneration and herniation, spinal stenosis, etc.^1^, ^2^ The fusion of the lumbar spine, however, may induce alterations in its inherent physiological structure, thereby resulting in a diminished physiological functionality. Consequently, this can significantly augment the load on neighboring intervertebral discs and facet joints, to degeneration of the adjacent segment.^3^, ^4^ Postoperative complications such as pseudoarthrosis, non‐union and internal fixation failure are more common in some patients.^5^, ^6^ Even if the fusion procedure achieves success, the patient's quality of life (QoL) may still be adversely affected by persistent discomfort in the lower back or leg. This can significantly impact their overall health and well‐being. The primary factor contributing to this issue is attributed to the excessive rigidity of the pedicle static fixator. Consequently, in order to effectively address this concern, there has been a development and widespread utilization of pedicle‐based stabilization (PDS) devices.^7^, ^8^ It provides sufficient stability to restore regular segmental movements, prevent instability, and minimize degeneration in adjacent segments.

The Dynesys dynamic stabilization system preserves intervertebral movement by implanting non‐rigid elastic elements into the spine, maintaining flexibility.^9^, ^10^, ^11^, ^12^ A long‐term follow‐up study (The minimal duration of follow‐up was 72 months) of 38 patients by Zhang *et al*.^10^ showed favorable long‐term clinical and imaging results with Dynesys dynamic stabilized decompression. However, the Dynesys dynamic stabilization system has a limited range of applications and limits the patient indications. Lee *et al*.^9^ found that, in cases of late degeneration and severe instability, interbody fusion is still considered the preferred approach over Dynesys dynamic stabilization. The Isobar TTL system documented by French researchers Lavaste and Perrin in 1993, is a stabilizing system featuring a semi‐rigid structure.^23^ The Isobar TTL system incorporates a built‐in damper composed of a semi‐rigid titanium‐alloy rod, which effectively mitigates stiffness and restricts both axial and angular movements within the transition segments.^13^, ^14^, ^15^ A finite element result by Chen *et al*.^16^ found that the Isobar TTL system provided the effect of maximum allowable displacement (beyond peak axial stiffness) to reduce stresses in the pedicle and at the facet joint compared to Dynesys. The available studies demonstrate positive short‐term outcomes of the Isobar TTL dynamic fixation in terms of pain alleviation, improvement in QoL, and preservation of lumbar range of motion (ROM) at stable levels. The clinical results are considered satisfactory.^17^ However, long‐term studies are limited. Therefore, a retrospective study was conducted to: (i) evaluate the effectiveness of utilizing the Isobar TTL system for dynamic stabilization in individuals with lumbar degenerative diseases; and (ii) assess the long‐term efficacy of the Isobar TTL system. In this report, we present the clinical results. The clinical outcomes obtained were favorable and demonstrated long‐term efficacy.

## Methods

### Patient Selection

The present retrospective study included all patients who underwent surgical intervention for posterior lumbar decompression and Isobar TTL dynamic internal fixation between June 2010 and December 2016. The ethics committee of West China Hospital, Sichuan University, approved this study (No. 2022241).

The inclusion criteria for this study were as follows: (i) patients who were confirmed with diagnosis of degenerative spondylolisthesis, lumbar disc herniation and lumbar stenosis based on imaging and physical examination; (ii) during the presentation, all patients underwent unsuccessful conservative treatment for over 6 months. This included taking nonsteroidal anti‐inflammatory medications, attending physical therapy sessions, and receiving chiropractic manipulations; (iii) body mass index (BMI) were within normal limits; and (iv) no history of spine surgery.

Exclusion criteria included: (i) patients who had clear contraindications for surgery; (ii) patients diagnosed with metabolic bone disorders, tuberculosis, tumors or infections; (iii) patients with multi‐level degenerative lumbar disease, late degeneration, severe instability and lumbar degenerative spondylolisthesis ≧ Meyerding II°; (iv) patients with incomplete clinical follow‐up information or follow up duration less than 5 years after surgery; and (v) patients who exhibited adjacent segmental disc degeneration prior to surgery (with Kellgren–Lawrence grade >3 or Pfirrmann grade > 3). Ultimately, a total of 40 patients with comprehensive clinical data were enrolled.

### Surgical Technique

The patient was placed prone, followed by standard disinfection and draping procedures. An incision was made through the skin, subcutaneous tissue, and dorsal fascia to expose the spinal components. Bilateral facet joints at both upper and lower surgical levels were identified, exposed, and protected. Pedicle screws were then inserted and properly positioned. After a partial laminectomy and removal of the ligamentum flavum, gentle traction was applied to the dural sac and nerve root for nucleus pulposus extraction. Finally, Isobar TTL rods were introduced into place and securely connected to the screws. Apply pressure and secure the screw nut while removing the tail cap. An intraoperative C‐arm X‐ray confirmed the satisfactory positioning of pedicle screws and rods. Following verification of hemostasis, a standard procedure was followed to insert a drainage tube and close the incision. The drainage tube was removed within 72 h post‐surgery. Subsequently, patients were instructed to wear a soft lumbar brace for 3 months.

### Clinical Assessment

The visual analogue scale (VAS) and Oswestry disability index (ODI) were employed for the assessment of low back pain, leg pain, and neurological status preoperatively, at 1 week postoperatively, 3 months postoperatively, and during the final follow‐up the surgical procedure. The collection of all patients' follow‐up information was conducted through outpatient visits or telephone‐based follow‐ups.

### Radiographic Measurements

The radiographic parameters of segmental ROM, intervertebral space height (IH) in stabilized segments and the upper adjacent segments were assessed using static and lateral flexion/extension X‐rays. Measurements of IH and ROM were preoperative, at 1 week postoperatively, and during the final follow‐up visit. IH refers to the average height of the anterior, middle, and posterior intervertebral spaces. At the same time, ROM is determined by calculating the difference in segmental angulations between flexion and extension X‐rays.

One junior and one senior spine surgeon participated in the measurement of the imaging data, and if there existed a large difference in results between the two measurers, a third author participated and evaluated the measurements.

### Adjacent Segment Degeneration

A clinical determination of adjacent segment disease (ASD) was made by detecting one or more indications suggestive of ASD using X‐ray and MRI imaging conducted at the neighboring spinal level^18^: (i) a reduction in the IH exceeding 3 mm on anteroposterior X‐ray images; (ii) forward or backward of the vertebral body by a distance exceeding 3 mm can be observed on lateral radiographs; (iii) the presence of sagittal translation exceeding 3 mm or a change in intervertebral angle exceeding 10° on lateral flexion/extension X‐rays; and (iv) the progression of disc degeneration at grade 1 or higher according to the Kellgren–Lawrence. While the MRI‐based grading system for disc degeneration is a dependable tool,^19^ our evaluation of postoperative disc degeneration primarily relied on X‐rays due to limited utilization of MRI scans during long‐term follow‐up after surgery. Due to the frequent occurrence of ASD in the upper disc, this study focused solely on analyzing degeneration in that specific region. Patients were defined as having adjacent segment disease (ASDis) if they had symptoms such as low back pain, leg pain, and intermittent claudication associated with ASD during postoperative follow‐up.

### Statistical Analysis

Normally distributed data were tested using the Student's *t*‐test, in cases where the data did not follow a normal distribution, the Wilcoxon rank‐sum test was utilized instead. Percentages were used to represent categorical data, and comparisons between groups were analyzed using the chi‐square or Fisher exact tests. The difference in improvement from preoperatively to the final follow‐up assessment was evaluated through a paired *t*‐test. The inter‐observer reliability of radiographic measurements was assessed using the intra‐class correlation coefficient (ICC), and an ICC of ≥ 0.75 was considered to be of good reliability. Statistical analyses were performed using SPSS 26.0 (SPSS Inc., Chicago, IL, USA), with *p* values < 0.05 considered statistically significant.

## Results

### Patient Baseline Characteristics

Forty patients, with an average age of 44.65 (13.13) years, were included in the assessment after being followed up for an average duration of 79.37 (47–105) months. Among them, 24 (60%) were male and 16 (40%) were female. All imaging parameters were measured by two authors and the average ICC for all parameter measurements was 0.917.The preoperative radiographic evaluation confirmed degenerative spondylolisthesis in 14 patients (35%). Both groups exhibited no significant differences in age, BMI, gender, smoking habits, diabetes, hypertension, and follow‐up period. Table 1 presents the demographic and baseline features of the patients.

**TABLE 1:** Patient demographic data and clinical characteristics between spondylolisthesis and disc disease.

Characteristic	Overall	Stenosis or herniated disc	Spondylolisthesis	*p*‐value
Age, years	44.65 ± 13.13	46.38 ± 13.52	41.43 ± 12.19	0.260
Gender, *n* (%)				
Female	16 (40.00%)	11 (42.31%)	5 (35.71%)	0.685
Male	24 (60.00%)	15 (57.69%)	9 (64.29%)	
BMI, kg/m^2^	22.63 ± 3.52	23.04 ± 3.59	21.87 ± 3.38	0.317
Smoking (yes/no)	11/29	8/18	3/11	0.528
Diabetes (yes/no)	6/34	4/22	2/12	0.926
Hypertension (yes/no)	14/26	10/16	4/10	0.532
Follow‐up duration, months	79.37 ± 25.86	80.27 ± 27.69	77.71 ± 22.97	0.770

Abbreviation: BMI, Body mass index;

### Radiological Outcomes

**FIGURE 1:**
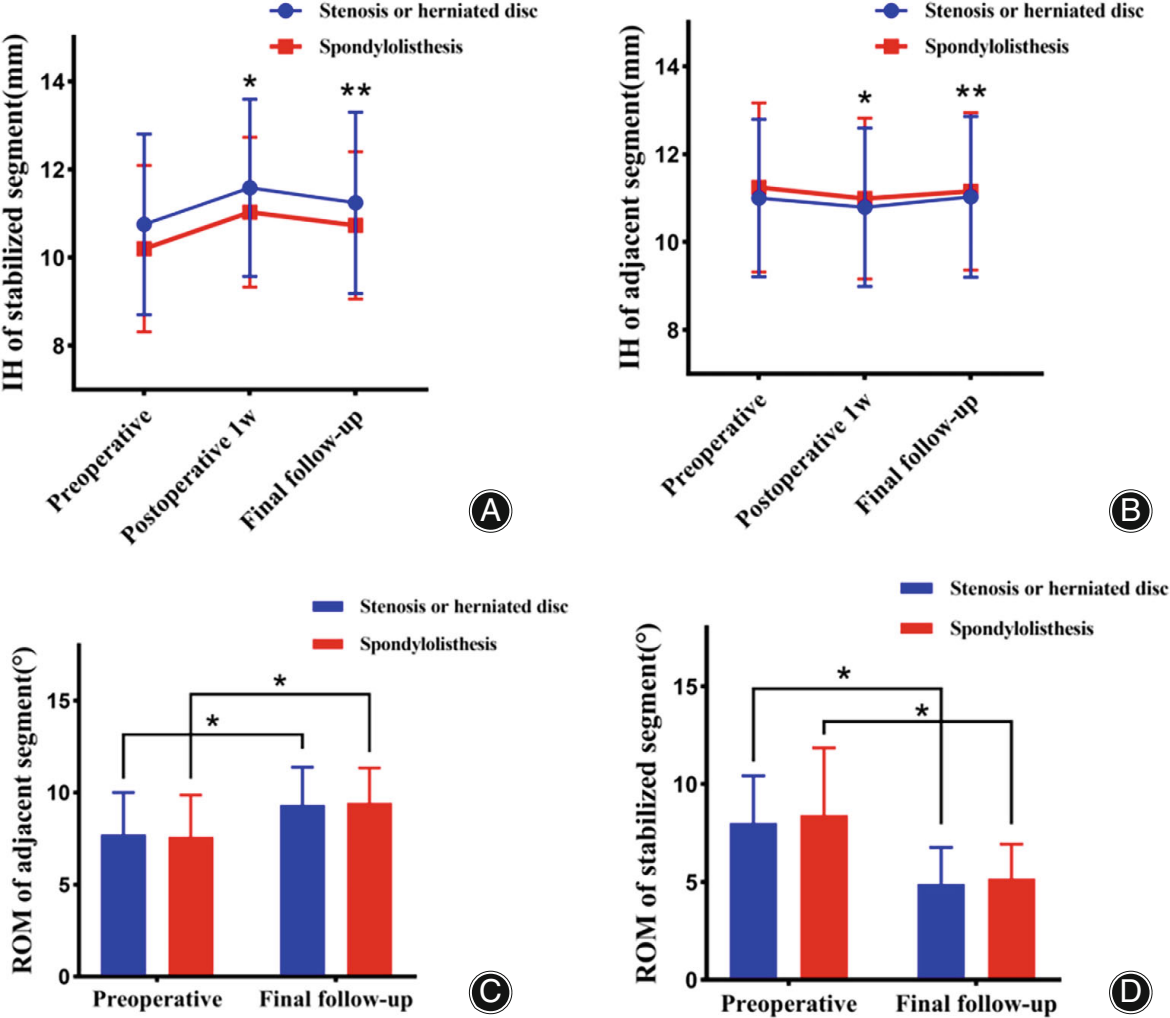
Longitudinal data describing the patient‐reported outcome measures IH of stabilized segment (A), IH of adjacent segment (B), ROM of adjacent segment (C) and ROM of stabilized segment (D) obtained preoperatively and during routine follow‐up postoperative. Smooth lines represent the mean patient‐reported outcome measure scores. Bars represent SD in both directions. ROM, range of motion; IH, intervertebral space height; SD, Standard deviation. *Comparison with preoperative, *p* < 0.001; **Comparison with postoperative 1w, *p* < 0.001.

**FIGURE 2:**
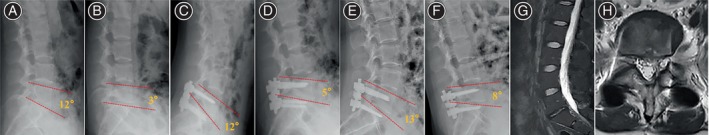
The radiological data of a patient with lumbar disc herniation. A 22 year‐old female patient underwent the Isobar TTL dynamic stabilization system due to lumbar disc herniation in L5/S1. The preoperative flexion and extension X‐rays (A, B) showed that the ROM of the stabilized segment was 9°. The flexion and extension X‐rays 3 months (C, D) after the operation, ROM of the stabilized segment was 7°. The flexion and extension X‐rays 9 years (E, F) after the operation that ROM of the stabilized segment was 5°. The MRI 9 years after the operation (G, H) showed normal disc signal intensity and no disc herniation at L5/S1 segment.

The ROM of the stabilized segment exhibited a significant decrease from 8.15° ± 2.77° prior to surgery to 5.00° ± 1.82° during the final follow‐up (*p* < 0.001; Table 2). Conversely, there was a slight increase in the ROM of the adjacent segment from 7.68° ± 2.25° preoperatively to 9.36° ± 1.98° postoperatively (*p* < 0.001; Table 2).There was no statistically significant difference in the postoperative ROM of stable segments and adjacent segments between the two groups. The IH of stabilized segments significantly increased from 10.56 ± 1.99 mm preoperatively to 11.39 ± 1.90 mm at the 1‐week follow‐up postoperatively (*p* < 0.001; Table 2).Subsequently, a slight decrease was observed in the average IH during the final follow‐up. Furthermore, the IH of adjacent segments exhibited a significant reduction from 11.09 ± 1.82 mm preoperatively to 10.86 ± 1.79 mm at the postoperative 1‐week follow‐up (*p* < 0.001; Table 2).The average IH slightly increased from 10.86 ± 1.79 mm at the 3‐month postoperative period to 11.07 ± 1.79 mm during the final follow‐up examination. However, no statistically significant differences were observed in ROM and IH between the stabilized and adjacent segments at any given time point for both groups (*p* > 0.05; Figure 1).The prevalence of ASD was 15% (6/40) after an average follow‐up duration of 79.37 months, with a rate of 15.38% (4/26) in the group with stenosis or herniated discs and 14.29% (2/14) in the spondylolisthesis group, The prevalence of ASDis was 5% (2/40), all occurring in the group with stenosis or herniated disc. No statistically significant difference was observed regarding the occurrence of ASD (*p* > 0.05). Throughout the follow‐up period, no severe complications such as loosening and breaking of the internal fixation, wound infection, or revision surgery related to treatment were reported, and none of the patients experienced moderate to severe intractable radiating pain. Figure 2 displays radiographs and MRI images from a representative patient.

**TABLE 2:** Summary of radiographic measurements between spondylolisthesis and disc disease.

Characteristic	Overall	Stenosis or herniated disc	Spondylolisthesis	*p*‐value
ROM of stabilized segment, °				
Preoperative	8.15 ± 2.77	8.01 ± 2.41	8.42 ± 3.43	0.658
Final follow‐up	5.00 ± 1.82	4.90 ± 1.87	5.17 ± 1.76	0.653
*P*‐value	<0.001	<0.001	<0.001	
ROM of adjacent segment, °				
Preoperative	7.68 ± 2.25	7.72 ± 2.28	7.59 ± 2.27	0.859
Final follow‐up	9.36 ± 1.98	9.33 ± 2.05	9.43 ± 1.91	0.886
*P*‐value	<0.001	0.001	0.001	
IH of stabilized segment, mm				
Preoperative	10.56 ± 1.99	10.75 ± 2.05	10.20 ± 1.89	0.408
Postoperative 1w	11.39 ± 1.90	11.58 ± 2.01	11.03 ± 1.70	0.394
Final follow‐up	11.06 ± 1.93	11.24 ± 2.06	10.73 ± 1.67	0.429
*P*‐value (post 1w *vs* preoperative)	<0.001	<0.001	<0.001	
*P*‐value (Final follow‐up *vs* post 1w)	<0.001	<0.001	<0.001	
IH of adjacent segment, mm				
Preoperative	11.09 ± 1.82	11.00 ± 1.79	11.24 ± 1.92	0.693
Postoperative 1w	10.86 ± 1.79	10.79 ± 1.80	10.99 ± 1.83	0.740
Final follow‐up	11.07 ± 1.79	11.03 ± 1.83	11.15 ± 1.79	0.843
*P*‐value (post 1w *vs* preoperative)	<0.001	<0.001	0.001	
*P*‐value (final follow‐up *vs* post 1w)	<0.001	<0.001	<0.001	
ASD^a^, *n* (%)				0.926
Yes	6 (15.00%)	4 (15.38%)	2 (14.29%)	
No	34 (85.00%)	22 (84.62%)	12 (85.71%)	

Abbreviations: ASD, adjacent segment degeneration; IH, intervertebral space height; ROM, Range of motion.

^a^ At final follow‐up.

### Clinical Efficacy

**FIGURE 3:**
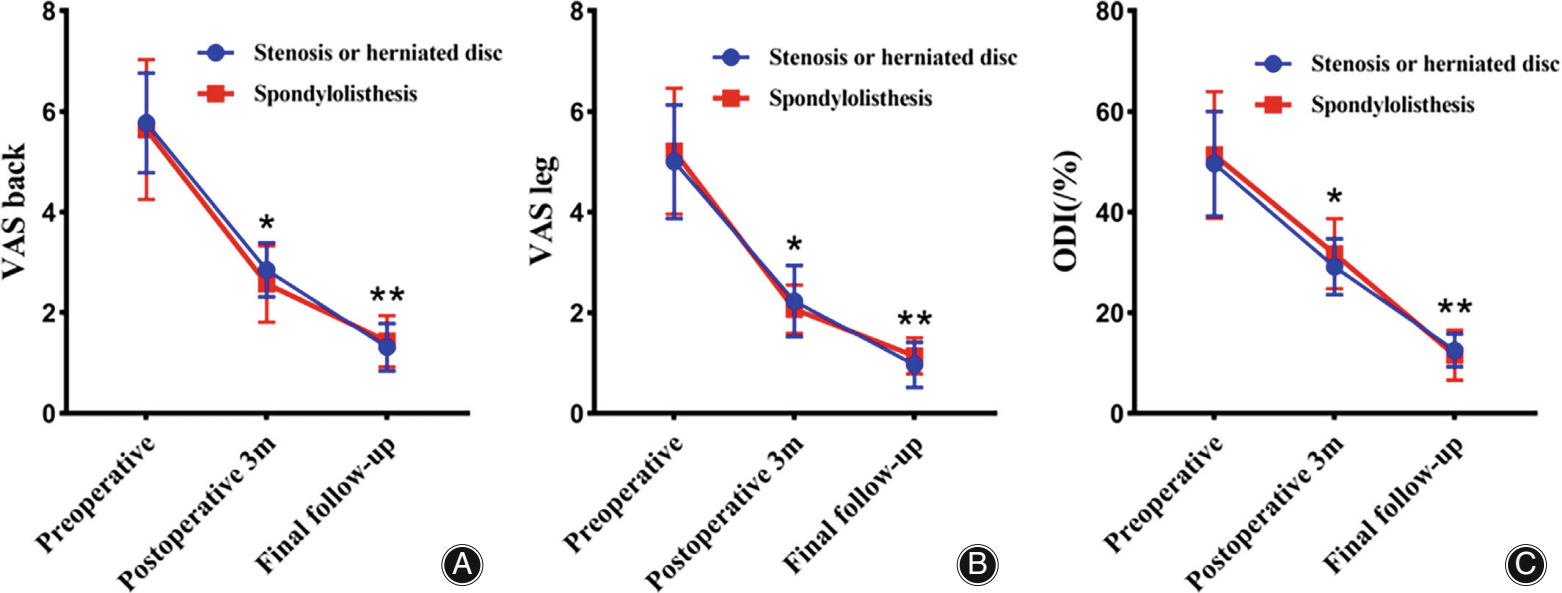
Longitudinal data describing the patient‐reported outcome measures VAS back (A), VAS leg (B), and ODI (C) obtained preoperatively and during routine follow‐up postoperative. Smooth lines represent the mean patient‐reported outcome measure scores. Bars represent SD in both directions. ODI, Oswestry disability index; VAS, visual analogue score; SD, Standard deviation. *Comparison with preoperative, *p* < 0.001;**Comparison with postoperative 3 m, *p* < 0.001.

The VAS scores for back and leg pain were significantly improved from 5.73 ± 1.13 and 5.08 ± 1.16 preoperatively to 2.75 ± 0.63 and 2.17 ± 0.64 at the 3‐month follow‐up postoperatively (*p* < 0.001; Table 3). The ODI scores were significantly improved at the 3‐month follow‐up postoperatively, as compared to the baseline values (50.23 ± 11.08 *vs* 30.03 ± 6.13, *p* < 0.001) (Table 3). Then, improvements in VAS and ODI scores continue long at the final follow‐up. The VAS scores for back and leg pain at the final follow‐up were 1.35 ± 0.48 and 1.02 ± 0.42, respectively. The ODI scores improved significantly from 30.03 ± 6.13 at the 3‐month postoperatively to 12.18 ± 3.92 at the final follow‐up. There were notable enhancements observed in both cohorts regarding VAS scores for back and leg pain and ODI scores. However, no significant disparities were found in the VAS for back and leg pain and ODI scores at any given time point between the two groups (*p* > 0.05; Figure 3).

**TABLE 3:** VAS and ODI scores between the two groups.

Characteristic	Overall	Stenosis or herniated disc	Spondylolisthesis	*p*‐value
VAS back				
Preoperative	5.73 ± 1.13	5.77 ± 0.99	5.64 ± 1.39	0.741
Postoperative 3 m	2.75 ± 0.63	2.85 ± 0.54	2.57 ± 0.76	0.192
Final follow‐up	1.35 ± 0.48	1.31 ± 0.47	1.43 ± 0.51	0.457
*P*‐value (Post 3 m *vs* Preoperative)	<0.001	<0.001	<0.001	
*P*‐value (final follow‐up *vs* post 3 m)	<0.001	<0.001	<0.001	
VAS leg				
Preoperative	5.08 ± 1.16	5.00 ± 1.13	5.21 ± 1.25	0.585
Postoperative 3 m	2.17 ± 0.64	2.23 ± 0.71	2.07 ± 0.48	0.457
Final follow‐up	1.02 ± 0.42	0.96 ± 0.45	1.14 ± 0.36	0.175
*P*‐value (post 3 m *vs* preoperative)	<0.001	<0.001	<0.001	
*P*‐value (final follow‐up *vs* post 3 m)	<0.001	<0.001	<0.001	
ODI, %				
Preoperative	50.23 ± 11.08	49.62 ± 10.39	51.36 ± 12.60	0.642
Postoperative 3 m	30.03 ± 6.13	29.12 ± 5.55	31.71 ± 6.98	0.205
Final follow‐up	12.18 ± 3.92	12.50 ± 3.28	11.57 ± 4.97	0.481
*P*‐value (post 3 m *vs* preoperative)	<0.001	<0.001	<0.001	
*P*‐value (final follow‐up *vs* post 3 m)	<0.001	<0.001	<0.001	

Abbreviation: ODI, Oswestry disability index.

## Discussion

This is a retrospective study to evaluate the effectiveness of utilizing the Isobar TTL system for dynamic stabilization in patients with lumbar degenerative disease. The results showed significant improvements in ODI and VAS scores for low back pain and leg pain in patients with and without spondylolisthesis compared with baseline scores at an average follow‐up of 79.37 months. Meanwhile, the Isobar TTL system preserved the ROM of the stabilized segments from 8.15° ± 2.77° preoperatively to 5.00° ± 1.82°. The IH of the operated segments was well maintained, with an increase in the IH of the adjacent segments. The prevalence of ASDs and ASDis was significantly reduced. Therefore, the clinical results of dynamic stabilization with the Isobar TTL system in patients with lumbar degenerative diseases were favorable and effective in the long‐term.

### Application of the Isobar TTL in Lumbar Degenerative Disease

The prevalence of lumbar degenerative disease as a cause of chronic back and leg pain among older individuals is substantial.^20^, ^21^ Its incidence increases with age and significantly impacts the QoL for seniors. Presently, posterior lumbar interbody fusion (PLIF) and transforaminal lumbar interbody fusion (TLIF) are widely acknowledged as the preferred surgical interventions for various degenerative lumbar pathologies.^1^, ^22^ However, the existing body of evidence suggests that adjacent segment degeneration may occur due to increased stress on the intervertebral disc and facet joint following spinal fusion.^3^, ^4^, ^5^, ^6^ To address this issue and effectively relieve patients' clinical symptoms, various spinal non‐fusion techniques with dynamic stabilization and internal fixation have been developed.

The Isobar TTL system, which was initially documented by Perrin in 1993, comprises a universal pedicle screw and two dynamic rods.^23^ The key component of this system is the dynamic rod, which is an exclusive shock‐absorbing joint constructed with internally stacked titanium rings.^24^, ^25^, ^26^ The shock‐absorption element exhibits an elastic range of motion that closely resembles the natural movement of the spine. It effectively absorbs shocks by allowing 2 mm three‐dimensional range of motion within ±2°. The Isobar TTL dynamic fixation system is designed to provide stability during the treatment of IDD while still preserving some degree of mobility in the treated segment. Isobar TTL has been reported to be particularly suitable for: (i) patients with lumbar disc herniation who are obese, heavy manual workers and have large herniated discs; (ii) patients with lumbar spinal stenosis who have central spinal stenosis, foraminal stenosis and lateral saphenous stenosis bilaterally; and (iii) patients with degenerative lumbar spondylolisthesis and instability. Most patients have reported satisfactory clinical outcomes. Li *et al*.^27^ conducted a retrospective analysis on 37 patients who had lumbar degenerative diseases and were treated with the Isobar TTL system, which is a semi‐rigid pedicle‐screw stabilization system. The findings from a period of 24 months indicated that implementation of the Isobar TTL system led to improvements in pain relief, QoL, functional capacity, and overall patient satisfaction. Gao *et al*.^24^ showed that the Isobar TTL system significantly improved preoperative JOA and ODI scores and was not different from patients undergoing posterior lumbar fusion.

### Long‐term Efficacy of Isobar TTL

Similarly, the study conducted by Qian *et al*.^17^ demonstrated that the Isobar TTL dynamic fixation system provided significant pain relief, improved QoL, and maintained ROM in patients with LDDs at an average follow‐up of 18 months. The achieved clinical outcomes were considered satisfactory. However, the follow‐up time was short in a few studies, and long‐term efficacy remains to be seen and should be further investigated. This study showed significant improvements in ODI and VAS scores for low back pain and leg pain compared with baseline scores in the patients with and without spondylolisthesis at an average follow‐up of 79.37 months. This finding is in agreement with previous data.^17^, ^24^, ^27^ Hence, we conclude that the Isobar TTL system might represent a therapeutic alternative against lumbar degenerative diseases.

The Isobar TTL is a dynamic fixation system designed for the lumbar spine, which involves inserting a flexible rod into the intervertebral space and fusing it with the vertebral arch. This innovative approach enables unrestricted movement of the lumbar spine, distinguishing it from conventional fusion techniques that may restrict normal vertebral motion. Compared to traditional lumbar fusion surgery, the Isobar TTL system has the ability to maintain a specific ROM in the stabilized segment and preserve the natural lumbar curvature, thereby ensuring ample spinal stability and preventing degeneration of adjacent segments. However, considerable controversy exists regarding preventing degeneration at adjacent segments, which may be related to the numerous factors that influence degeneration. ASD is usually not clinically significant, and surgical decompression is an option for the few patients with symptoms. Several factors affect ASD, and previous studies have found that the main risk factors include age, BMI, and the degree of preoperative adjacent segment degeneration. Consistent with previous studies, there was no significant difference in the incidence of ASD between lumbar spondylolisthesis and lumbar disc herniation or spinal stenosis. However, many research studies have demonstrated the efficacy of the Isobar TTL system in maintaining ROM stability and mitigating radiological ASD. Our study showed that the Isobar TTL system preserved ROM at the stabilized segments from 8.15° ± 2.77° preoperatively to 5.00° ± 1.82°, at the same time, as with the findings of Guan *et al*.^13^ at a mean follow‐up of 52.23 months, the IH in the operated segment of Isobar EVO and Isobar TTL was well maintained while in the adjacent segment was increased. Therefore, we believe that the Isobar TTL system disperses a portion of the stresses placed on the adjacent segments, while a certain degree of ROM maintenance contributes to disc rehydration, which is more effective in reducing the incidence of ASD.^28^ According to Zhang *et al*.,^29^ a comparison was made between lumbar fusion patients and the Isobar TTL system, which revealed that the utilization of the Isobar TTL system effectively prevented adjacent segment degeneration as well as screw breakage.

Similarly, Korovessis *et al*.^30^ and Hrabálek *et al*.^31^ achieved the same conclusion. This research discovered that the occurrence of ASD following the implementation of the Isobar TTL system was 15%, and the ASDis was 5%. The prevalence of ASD and ASDis is significantly lower in comparison to lumbar fusion reported in the literature. In contrast, Li *et al*.^27^ and Fu *et al*.^32^ concluded that the Isobar TTL semi‐rigid fixation system was ineffective in preventing adjacent segment degeneration. In a prospective study with a 24‐month follow‐up, Fu *et al*. discovered that patients underwent significant clinical improvement. However, there appeared to be ongoing disc degeneration at both the stabilized and adjacent segments: the average Pfirrmann score increased slightly from 2.86 before surgery to 2.92 after 24 months at the stabilized segment, from 1.92 preoperatively to 1.96 after 24 months at the adjacent segment. However, the preoperative and postoperative Pfirrmann scores did not show any statistically significant difference. This raises questions about the conclusion made by Fu *et al*. regarding the ineffectiveness of the Isobar TTL semi‐rigid fixation system in preventing adjacent segment degeneration. Although none of the patients in this series required revision surgery, further research with a larger sample size and longer follow‐up periods is necessary to validate the protective effect of the Isobar TTL system on ASD.

### Limitations and Strengths

This is the first study to evaluate the long‐term outcome of the patients treated with the Isobar TTL dynamic stabilization system for lumbar degenerative diseases. This study demonstrated that the Isobar TTL system was effective in maintaining ROM in stabilized segments; maintaining IH in operated segments, and significantly reducing the prevalence of ASD and ASDis. The clinical results of dynamic stabilization with the Isobar TTL system in patients with lumbar degenerative diseases are favorable and effective in the long‐term.

However, it is essential to acknowledge the limitations of our study. First, this retrospective study was conducted in a single center with a relatively small sample size. It would be beneficial to conduct further large‐scale prospective randomized controlled trials to validate our findings. Second, it should be noted that no control group was included in our study design, adding a control group with conventional lumbar interbody fusion and rigid internal fixation could provide stronger evidence to support the long‐term efficacy of Isobar TTL. Despite these constraints, our data provides additional evidence supporting the long‐term effectiveness of the Isobar TTL system in treating lumbar degenerative diseases. Third, although there is some consensus on the limited use of dynamic fixation techniques such as Isobar TTL in patients with late degeneration and severe instability of the lumbar spine, and our study excluded patients with severe degeneration and instability, relevant long‐term follow‐up studies are still needed. Finally, as a long‐term follow‐up study, more follow‐up time points such as 6 months postoperatively, 1 and 2 years postoperatively may provide more information.

## Conclusions

The Isobar TTL dynamic fixation system for LDDs showed satisfactory long‐term clinical and radiographic results, which might represent a therapeutic alternative against lumbar degenerative diseases and showed its great potential in avoiding adjacent segment degeneration.

## Conflict of Interest Statement

The authors confirm that they have no conflict of interest with respect to the manuscript content or funding.

## Ethics Statement

This retrospective study was in accordance with the ethical standards of the institutional and national research committee and with the 1964 Helsinki Declaration and its later amendments or comparable ethical standards. The ethics committee of West China Hospital, Sichuan University approved this study (No. 2022241).

## Author Contributions

All authors contributed to the study conception and design. Material preparation, data collection and analysis were performed by Junhu Li, Qiujiang Li and Linnan Wang. The first draft of the manuscript was written by Junhu Li and all authors commented on previous versions of the manuscript. All authors read and approved the final manuscript.

## Authorship Declaration

All authors listed meet the authorship criteria according to the latest guidelines of the International Committee of Medical Journal Editors and all authors are in agreement with the manuscript.

## Informed Consent

The information relating to the patients are completely anonymized and the submission does not include images that may identify the person.
